# A Deep-Learning Framework for Analysing Students' Review in Higher Education

**DOI:** 10.1155/2023/8462575

**Published:** 2023-03-16

**Authors:** Blessings Ngwira, Baby Gobin-Rahimbux, Nuzhah Gooda Sahib

**Affiliations:** Department of Software and Information Systems, Faculty of Information Communication and Digital Technologies, University of Mauritius, Reduit, Mauritius

## Abstract

As part of continuous process improvements to teaching and learning, the management of tertiary institutions requests students to review modules towards the end of each semester. These reviews capture students' perceptions about various aspects of their learning experience. Considering the large volume of textual feedback, it is not feasible to manually analyze all the comments, hence the need for automated approaches. This study presents a framework for analyzing students' qualitative reviews. The framework consists of four distinct components: aspect-term extraction, aspect-category identification, sentiment polarity determination, and grades' prediction. We evaluated the framework with the dataset from the Lilongwe University of Agriculture and Natural Resources (LUANAR). A sample size of 1,111 reviews was used. A microaverage *F*1-score of 0.67 was achieved using Bi- LSTM-CRF and BIO tagging scheme for aspect-term extraction. Twelve aspect categories were then defined for the education domain and four variants of RNNs models (GRU, LSTM, Bi-LSTM, and Bi-GRU) were compared. A Bi-GRU model was developed for sentiment polarity determination and the model achieved a weighted *F*1-score of 0.96 for sentiment analysis. Finally, a Bi-LSTM-ANN model which combined textual and numerical features was implemented to predict students' grades based on the reviews. A weighted *F*1-score of 0.59 was obtained, and out of 29 students with “*F*” grade, 20 were correctly identified by the model.

## 1. Introduction

Tertiary institutions play a significant role in equipping human resources with specialized skills necessary for improving the social-economic growth of a country. Raising the bar in teaching and learning is paramount in producing quality graduates capable of transforming a nation's economic status. Achieving this requires investing in quality assurance activities for the different aspects of teaching and learning. Factors that affect the quality of knowledge acquisition by students in tertiary institutions include students' learning styles, instructors' teaching methodology, preparedness knowledge, learning environments, and teaching and learning resources. For instance, different students prefer different learning styles to understand what is being taught and this may result to significant differences [1, 2]. Against this background, it is therefore important for instructors to align their delivery methods with students' preferred learning to effectively transfer knowledge.

As part of quality assurance activities, most tertiary institutions collect students' module feedback data at the end of each semester. The feedback contains students' ratings for their instructors on several attributes such as preparedness, teaching skills, availability, and behavior. Teaching and learning resources such as laboratory equipment and classroom ventilation are also assessed. The management of tertiary institutions leverages insights drawn from analyzing this feedback data to make informed decisions. Preventative and corrective measures are then taken to address the different challenges encountered by students during the semester. This not only improves the quality of teaching and learning but also increases learner satisfaction.

Manually analyzing thousands of students' qualitative comments is inefficient for the following reasons. Firstly, it may lead to subjective and inconsistent interpretations, thus reducing the reliability and repeatability of the results. Secondly, this approach is prone to human errors arising mostly due to fatigue when carrying out repetitive tasks manually. Thirdly, it reduces staff productivity levels considering that a significant amount of effort and time needs to be allocated to this task. Otherwise, given the right tools, the human resource allocated to performing this task could be freed and efficiently utilized for other crucial activities of the organization. Fourthly, with the manual approach, it is difficult to mine hidden insights from the massive volume of students' feedback. Keeping track of recurring themes and patterns from students' past feedback is a daunting task. Thus, valuable insights are left untapped. Furthermore, it is challenging to predict the academic performance of students prior to writing final examinations.

Innovative and cost-effective approaches are needed by tertiary institutions to analyze the reviews effectively and efficiently. Recently, there has been a surge in research in the fields of natural language processing (NLP) and machine learning. These techniques can be leveraged in mining insights from the reviews. However, to ensure consistency, reliability, credibility, and accuracy of the analysis, it is essential to formulate a roadmap for the analysis. Therefore, this study proposes a novel framework for analyzing students' module feedbacks. The framework leverages existing NLP and deep learning techniques.

The aim of the research is to create a framework for analyzing students' module feedback. It is expected that the outcome of the reviews, based on the proposed framework will assist the management of tertiary institutions to efficiently and effectively mine valuable insights from students' qualitative module reviews. With the insights extracted from the analysis of the reviews, university management can make informed decisions on improving teaching and learning processes. In this paper, three models were used for aspect term extraction, namely, LSTM-CRF, GRU-CRF, and Bi-LSTM-CRF. For the task of aspect category identification, four RNN variants (LSTM, GRU, Bi-LSTM, and Bi-GRU) were built and compared. Based on the aspect terms, the sentiment polarity of the students' reviews was determined. At the institution under study in this paper, students' feedback is collected prior to the examination. Therefore, a grade prediction model was also developed in order to identify which students are likely to fail based on their review of the module. In this respect, course leaders can develop initiatives to further support these students.

The rest of this paper is structured as follows. In Section 2, existing works in this domain are presented; in Section 3, the different steps in the developed framework for analyzing students' reviews are described. The findings of this study are discussed in Section 4, and Section 5 concludes the study, outlining possibilities for future works.

## 2. Literature Review

In this section, we describe aspect-based sentiment analysis and recent works in analyzing students' reviews through NLP and deep learning techniques.

### 2.1. Sentiment Analysis

Sentiment analysis is defined as the process of mining people's opinions from [3]. The opinions can be categorized as either positive, negative, or neutral. Sentiment analysis can be performed at three levels of granularity: document, sentence, or aspect level [4]. At the document level, sentiment analysis is performed for the entire text document. The document is classified into a positive, negative, or neutral class [5]. Document level sentiment analysis is too generic since it does not provide polarity scores for the individual reviews contained in the document. Hence, it is a shallow analysis that just provides an overview. Sentence level sentiment analysis is a deeper analysis compared to the document level. It is performed at sentence granularity. Thus, sentiment polarity scores for each individual sentence in the document are calculated. This analysis assumes that there is a single opinion in the sentence which is not usually the case in the real world. For example, a student might say “the lecture was great, but the classroom size was too small to accommodate all the students [6].” This sentence has got two different opinions: “great” directed onto “lecture” and “too small” expressed on “classroom size.” Sentiments for these two opinions need to be captured separately hence the need for a much finer granular analysis.

Aspect-based sentiment analysis (ABSA) is a subdiscipline of NLP. It pays special attention to sentiments target known as aspects in a sentence [7]. It is more granular than document and sentence level sentiment analysis and hence more complex to implement [8]. The aspect-based sentiment analysis (ABSA) task is to identify opinions expressed toward specific entities or attributes [9]. ABSA consists of the following three main tasks [10, 11], namely, (1) identification of aspects from the text; (2) extraction of the linguistic expression used to refer to the aspects so as to identify entities and attributes; and (3) identification of the sentiment polarity or opinion of each aspect. ABSA is often used to identify the sentiment polarity or opinion in customer reviews for e-commerce websites and tweets [11] but has also been previously used in education-related fields for various purposes: to review the performance of teaching staff [12] and to review students' social media posts [13].

There are different approaches for sentiment analysis, namely, lexicon, “traditional” machine learning, and deep learning approaches. Lexicon approaches are dictionary-based. The dictionary contains a collection of sentiment terms and their associated polarity values [14]. With the lexicon approach, words are assigned sentiment polarity values based on the dictionary mappings between words and sentiment polarity scores. However, if a word is not found in the dictionary, its sentiment polarity score cannot be determined. Since the dictionary mapping does not capture the context of the sentiment terms, lexicon approaches do not scale well with words that have contrasting sentiment polarity scores in different domains or contexts. Popular lexicon dictionaries are Sentiword, AFINN, VADER, SentiwordNet, TextBlob, and OpinionFinder [4].

Machine learning approaches to sentiment analysis treat sentiment analysis as a classification problem [14]. Recent trends in sentiment analysis are leveraging the capabilities of deep learning. This is because deep learning models are capable of identifying complex relationships and patterns from datasets. Sentiment analysis models constructed using deep learning require less feature engineering compared to traditional machine learning approaches. Recurrent neural networks (RNNs) and long short-term memory (LSTM) networks are usually applied to sequential tasks such as sentiment analysis, named entity recognition, speech processing, DNA sequences, and machine translations [6]. The architecture for RNN allows a neural cell to learn from previous time step outputs [9, 15, 16]. This capability makes it possible for RNNs to learn from the past to improve future performance. A major disadvantage of RNNs is that they suffer from the problem of vanishing gradients, especially for long sentences [17]. LSTM and gated recurrent units (GRUs) were specifically designed to deal with this problem. Their architectures contain gates that control which information to retain or remove from the memory. Another challenge with RNNs is that they are limited to only learning from previous time steps only. To address this problem, bidirectional RNNs were introduced. Bidirectional RNNs contain two layers of RNNs that process inputs in opposite directions. Outputs from the two RNNs are concatenated and forwarded to the next layer [6]. This ensures that the neural network learns from both forward and backward propagation Variants of bidirectional RNNs are Bi-LSTM and Bi-GRUs which also operate in a similar manner with the added advantage of not having the vanishing gradient problem faced by RNN.

### 2.2. Aspect-Based Sentiment Analysis of Students' Reviews

Aspect-based sentiment analysis (ABSA) is a subdiscipline of natural language processing. It pays special attention to sentiments target is known as aspects in a sentence and is more complex [7, 8]. ABSA identifies opinions expressed towards specific entities or attributes [9] and consists of the following three main tasks: (1) identification of aspects from the text, (2) extraction of the linguistic expression used to refer to the aspects so as to identify entities and attributes, and (3) identification of the sentiment polarity or opinion of each aspect [10, 11]. ABSA is often used to identify the sentiment polarity or opinion in customer reviews for e-commerce websites and tweets [11].

Chauhan et al. [13] did an ABSA at the document level to review students' posts on social media. Nouns, noun phrases, and an ontology containing concepts of higher education was used for aspect extraction. The authors used a naive-based classifier to do the sentiment analysis. Shaikh and Doudpotta [18] used ML and a rule-based approach to develop a sentence-level ABSA system. SentiWordNet was used for opinion mining and the corpus of the Pakistani language was manually labelled by the authors. Naive Bayes multinomial classification was used for entity extraction.

Sindhu et al. [19] used LSTM to do sentence-level ABSA to detect all aspects and sentiment polarity. A custom word embedding was developed. 5000 surveys from the Sukkur IBA University and the SemEval 2014 corpus for a noneducational domain were used. The *F*1-score was 93% for sentiment orientation detection and 91% for aspect extraction.

Nikolić et al. [7] implemented ABSA on reviews within the higher education domain. They developed a system for ABSA on reviews written in the Serbian language using NLP techniques and machine learning models at the sentence segment level. The author did not use any deep learning model due to a lack of data. They used two classification methods: first, an SVM model and the second one is based on dictionary matching.

Kastrati et al. [20] first used conventional machine learning techniques to classify the polarity of a review based on aspects as well as a 1D-CNN model using various word embeddings. The authors then developed an LSTM and CNN deep learning model for aspect category sentiment classification [21]. They used the FastText, GloVe, Word2Vec, and domain-specific MOOC word embeddings for the experimentation. The systematic literature review carried out by [22] reviews existing work which discuss sentiment analysis of student's feedback. The author's classified the various papers into three categories: (1) a set of papers which deal with comments where the aspects focus on teacher entity, which include (teacher's knowledge, pedagogy, and behavior); (2) papers discussing aspects on courses, teachers, and institutions; and (3) papers dealing with capturing the opinions and attitudes of students toward institution entities.

## 3. Methods and Results

**Figure 1:**
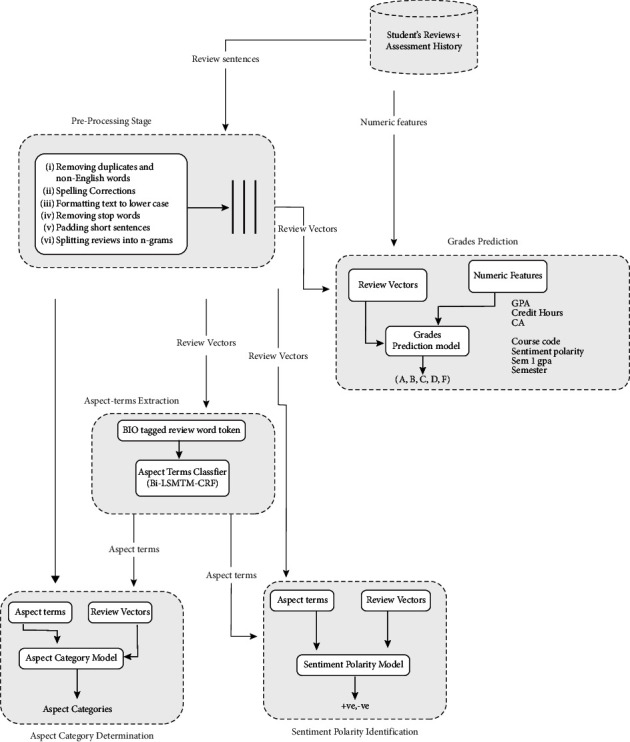
Framework for analyzing students' module feedback.

In this section, the methodology used to analyze students' reviews, determine the sentiment polarity, and predict grades are explained as shown in Figure 1.

### 3.1. Data Acquisition

The first stage of data acquisition was domain understanding. The objectives set at this stage were two-fold: firstly, defining aspect categories found in students' reviews and secondly, labelling training and testing datasets. These objectives were achieved by working collaboratively with education domain experts. With the realization that expert knowledge might be biased, the task of labelling the dataset was performed independently by two education domain experts. Inconsistencies observed between the two were resolved through consensus. At the end of this stage, a list of aspect categories was produced. An aspect category was defined based on the frequency of related comments in the review. With assistance from the education domain experts, the categories were labelled.  Table 1 lists selected phrases associated with each aspect category.

The students' module reviews dataset is from the Lilongwe University of Agriculture and Natural Resources, a public university in Malawi. The dataset consists of records which were obtained for a period of four consecutive academic years. In each academic year, students' modules feedback was captured at the end of every semester prior to writing examinations. The dataset contains both quantitative and qualitative data. The university has 5 faculties and an average enrolment of 4000 students each academic year.

### 3.2. Data Preparation and Preprocessing

Once the dataset was acquired, duplicates, non-English characters, and misspellings were removed from the dataset. Stop words and punctuation marks were also removed. The aspect terms in each individual review were manually labelled using the BIO tagging scheme. In order to ensure efficient processing of the dataset by machine learning algorithms, the following standard preprocessing steps were carried out: converting all texts to lowercase, spelling correction, padding short sentences, tokenization, and vectorization. All the reviews were converted to vectors which were then used to build the grade prediction and for sentiment analysis. The review vectors were also used for aspect-term extraction and aspect category determination. The resulting aspect terms obtained were used for sentiment analysis.

### 3.3. Aspect-Term Extraction and Modelling

Aspect terms were labelled with either “B-tag” or an “I-tag.” If an aspect term consisted of two or more words, the first word was assigned a “B-tag,” whereas all the remaining words in that compound aspect terms were given an “I-tag.” Aspect terms comprising of a single aspect word were labelled with a “B-tag.” If a word was not part of the aspect terms, it was given an “O-tag” as shown in Table 2. The “B-I-O” tagging annotation is summarised in Table 3. SemEval datasets act as a de facto standard for ABSA and the guidelines were used to the data annotation process. The aspect terms, aspect categories, and sentiment polarity classes were labelled. Aspect terms are attributes of entities upon which sentiment expressions are directed in a given review sentence [23]. For example, consider a review sentence R: “the number of students in our class is very large.” In this case, “number of students” constitutes aspect terms. Usually, aspect terms are nouns or noun phrases. Training and testing dataset for the aspect's extraction model were labelled using the BIO Tagging since aspect-term extraction is considered as a sequence labelling task [10].  Table 4 shows the number of reviews used as the training and test data and the number of B-tags, I-tags, and O-tags.

The goal of the aspect-term model was to extract aspect terms from students' qualitative reviews. Aspect terms as defined by [23] as entity attributes on which review sentiments are directed onto. Given a sequence of review words, the task was to extract aspect terms found in the sequence using the BIO tagging scheme.

**Figure 2:**
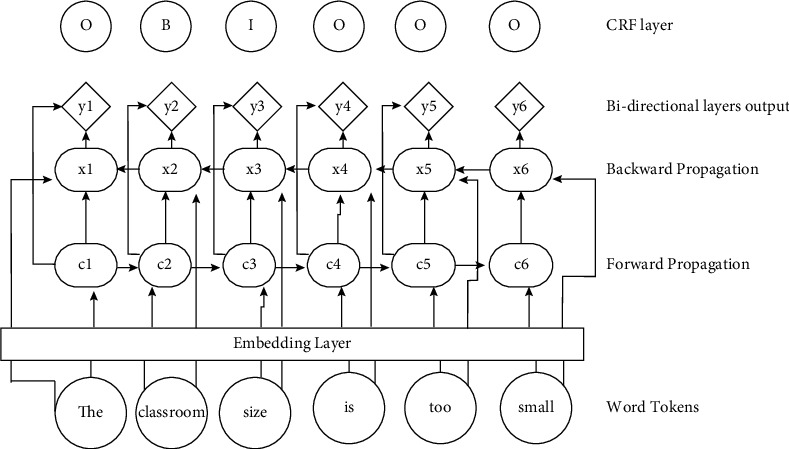
Bi-LSTM and CRF architecture for aspect-term extraction.

Figure 2 below shows Bi-LSTM-CRF architecture which was implemented to extract aspect terms from the students' reviews. POS vectors and word vectors were fed as inputs via the word embedding layer into the Bi-LSTM neural network. Outputs from the Bi-LSTM layer were then passed onto a final CRF layer. The CRF layer concatenated output results from individual time steps of the Bi-LSTM neural network and using constraints it learned from the dataset guided the final predictions for the labels. This ensured that the entire sentence context was considered when predicting the final BIO-tagged labels. Based on previous works, three models were implemented, namely, Bi-LSTM-CRF, LSTM-CRF, and GRU-CRF neural networks.

Table 5 shows the results found after experimenting with the three models. The Bi-LSTM-CRF model achieved the highest micro- and macro-*F*1-scores of 0.67 and 0.66, respectively. A micro-*F*1-score of 0.67 recorded by the Bi- LSTM-CRF model implies that an overall accuracy of 67% can be reached when predicting “B” and “I” aspect terms from the dataset using the model. Furthermore, a consistent pattern of higher *F*1-scores for “B” aspect term compared to “I” aspect term can be observed in all the three models from Table 4. This trend could be explained by the difference in sample size representations of “I” and “B” aspect terms in both the training and testing sets. Our dataset had more “B” aspect term compared to “I” aspect term considering that most of the reviews had a single aspect term in them. From Table 4, it can also be revealed that a 0.74 *F*1-score for the “B” aspect term using Bi-LSTM-CRF classifier was recorded.

On the other hand, a 0.53% *F*1-score for “I” aspect term using the Bi-LSTM-CRF classifier was achieved. One possible explanation for the high performance achieved by the Bi- LSTM-CRF classifier compared to the rest of the models is because of its internal architecture which enabled it to learn from both forward and backward propagations hence improving its performance.

### 3.4. Aspect-Category Identification Model

Several studies have been conducted in tackling the problem of aspect-category detection in literature. Akhtar et al. [24] approached this task as a supervised multilabel classification problem. Mamatha et al. [9] identified seed words or aspect terms associated with each category and leveraged them when predicting the categories. Using a conditional random field classifier together with grammatical relationship dependencies, they were able to predict an aspect category for each review. Considering that different aspect categories contain different topics, Movahedi et al. [25] leveraged deep learning's attention mechanism in detecting aspect categories.

Based on previous works in this field, four variants of RNNs models were constructed and compared. LSTM, Bi-LSTM, GRU, and Bi- GRU models were trained on the labelled dataset and tested. Among the reasons for experimenting with variants of RNNs in performing this task was to utilize the capabilities of RNNs for processing sequential inputs. RNNs have proven to perform well in processing sequential tasks such as speech recognition, named entity recognition, and sentiment analysis. After comparing the performance of the different models, the model which achieved the highest performance was adopted and used for the rest of this study.

Since the aspect-category training and testing sets were uneven considering that some categories had fewer instances compared to other categories; the weighted *F*1-score metric was a better metric to use in evaluating our model performance because it considers class size when calculating it. From Table 6, it can be seen that three models: GRU, Bi-GRU, and LSTM achieved similar weighted *F*1-scores of 0.78, whereas LSTM achieved a slightly lower weighted *F*1-score of 0.77. Theoretically, it was expected that either Bi-LSTM or Bi-GRU models would achieve the highest performance considering that they process inputs from both forward and backward propagation. This enables them to update training weights based on concatenated outputs from the two opposite processing layers. A possible explanation for this deviation from the expected theoretical performance could be that our labelled dataset size was small for the benefits of bidirectional RNNs to be fully realized.

Since GRU, Bi-GRU, and LSTM models achieved similar performance in terms of weighted *F*1-score, it is recommended to use either of these three models depending on resource constraints and dataset size at hand. In environments where there are limited computational resources and the dataset is smaller, GRU neural networks are recommended because their architecture is computationally optimized to efficiently utilize resources during processing. For a large dataset, Bi-GRU is a better option. The 0.78 weighted average *F*1-score recorded by GRU, Bi-GRU, and LSTM models implies that one can be confident that the models would accurately predict 78% of aspect categories from the dataset.

It can also be seen from Table 6 that GRU, Bi-GRU, and LSTM models perform consistently well in predicting individual aspect categories as evidenced by *F*1-scores of at least 0.70 in more than seven categories out of twelve. On the other hand, it is also observed that there are variations in performance scores for the different aspect categories. This could be attributed to ambiguity caused by certain reviews that belonged to multiple aspect categories.

In comparison with the performance of state-of-the-art models for the task of aspect-category detection in the education domain, study findings conducted by [19] achieved the highest *F*1-score of 0.85. Among the reasons for the high score was the use of education domain word embeddings. Theoretically, using domain embedding enhances model performance. Despite the limitation of having a small labelled dataset, considering that deep learning models require massive amounts of training sets, the models developed in this work performed relatively well with respect to state-of-the-art models in predicting the aspect categories. The key for achieving this performance was the use of aspect terms as seed words that aided the neural networks to predict corresponding aspect categories for the students' reviews.

### 3.5. Sentiment Polarity Model

The goal for this model was to predict sentiment polarity scores for reviews with respect to aspect terms found in each review. Given a review sentence and an aspect term as inputs to the model, the model outputs either a negative or positive sentiment polarity score in the set {0, 1}. In this study, the authors focused on predicting positive and negative sentiments for the reviews and leaving out neutral ones. This was because most of the neutral comments were suggestions on how to improve various aspects of teaching and learning. As such neutral comments were treated as implicit negative sentiments considering that such suggestions implied that the present state had problems that required resolving.

Bi-GRU neural network was chosen as the model for two reasons, namely, to utilize its bidirectional processing capability that allows the neural network to adjust training weights based on updated information from both forward and backward propagation, hence improving performance. Secondly, Bi-GRU architecture consists of only two gates which is fewer than the Bi-LSTM's three gates. These gates control what information to store or discard from the neural network [26]. This makes it computationally less resource-intensive during training compared to Bi-LSTM neural networks.

To utilize aspect-term information in a review when predicting sentiment polarity, both sentence and aspect embeddings were used during model training. To the best of our knowledge, no study conducted so far in the education domain for sentiment analysis has experimented with the use of Bi-GRU with aspect embeddings in constructing a sentiment polarity classifier. Since there were no available open-source academic domain embeddings among the research community, Stanfold's Glove 840B was used to create word vectors in capturing word semantic meanings. The choice of Glove 840B was taken due to empirical evidence presented by [27]. Their findings revealed that Glove 840B and fastText embeddings achieved statistically superior performance compared with the rest of the embeddings.

The word vectors of sentences and the aspect terms' vectors were fed as inputs via sentence embedding and aspect embedding layers respectively. Sentence embeddings consist of semantic word representations for all word tokens in reviews, whereas aspect-term embeddings consist of semantic word representations for all aspect terms. Output from the two embedding layers was forwarded onto the Bi-GRU layers. The Bi-GRU layers processed the input sequences using both forward and backward propagation and then concatenated the results from the two bidirectional processing.

Considering that the dataset used was partially imbalanced since it had more negative reviews compared to positive ones in both the training and testing sets, the weighted *F*1-score was a better metric for evaluating the model performance since it considers class weights when computing it. As shown in Table 7, an overall *F*1-score of 0.96 was found from the average of 4 runs. Among the possible reasons for the high performance recorded in this study include the computational processing capability of Bi-GRU for sequential tasks using its bidirectional architecture. This allowed the neural network to learn from both forward and backward propagation, hence achieving superior performance. Aspect embedding information also played a significant role in determining sentiment polarity scores.

Thus, the Bi-GRU neural network performed considerably well in predicting sentiment polarity scores.  Table 8 gives an overview of the performance of the models compared to other models which have been implemented for sentiment polarity prediction within the education domain. Furthermore, out of 160 negative sentiments from the dataset, the proposed Bi-GRU model correctly predicts 158 of them as negative, and similarly, out of 31 positive sentiments, it correctly predicts 26 of them as positive.

### 3.6. Grades' Prediction Model

In this section, we investigate whether module reviews can be leveraged in predicting students' module grades. Predicting students' grades prior to writing examinations is important considering that it acts as an early warning sign in identifying students who are at risk of failing certain modules. After identifying the students at risk of failing, the management of the course can put in place preventative interventions to reduce failure rates. Normally, the reviews are conducted towards the end of each semester before students participate in the end-of-semester examinations.

**Figure 3:**
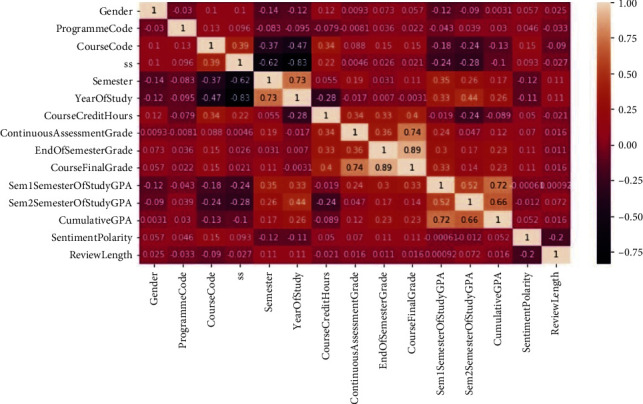
Heatmap for selection of variables.

To accomplish the task of predicting students' grades while considering both the students' reviews feedback and students' academic performance history, a multi-input Bi-LSTM-ANN neural network that takes as inputs both textual and numerical features is proposed. For the text features, the model takes in review sentence vectors via an embedding layer onto its Bi-LSTM neural network. The numerical features considered are continuous assessment grade, sentiment polarity score, CGPA, course credit hours, course code, first semester GPA, and semester. The numerical features were obtained after performing the heatmap variable selection technique on the dataset with respect to the target variable, that is, the course final grade. Numerical variables that achieved at least 0.1 correlation coefficient with the target variable were considered as feature candidates for the model as shown in Figure 3.

The text processing results from the Bi-LSTM neural network were concatenated with the numerical inputs from the ANN layer and passed to a dense neural network. The dense neural network extracted patterns from the combination of text and numerical features before forwarding the results to a final dense layer containing the SoftMax activation function. The SoftMax function generated probability distribution values for the five grade ranges {A, B, C, D, F}.

**Figure 4:**
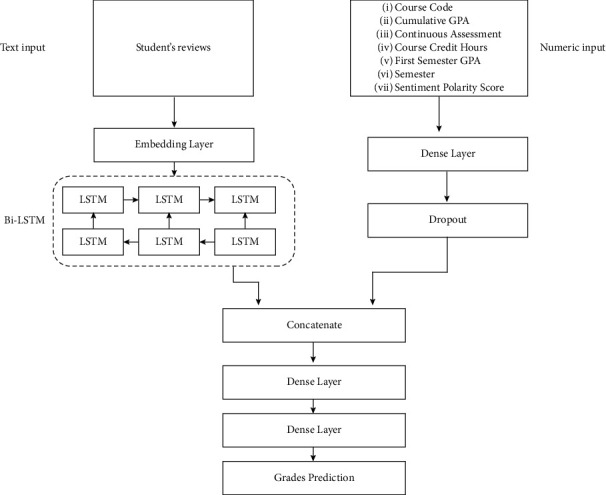
Proposed architecture for the grades' prediction model.

To evaluate the performance of the Bi-LSTM-ANN, its performance was compared with a baseline ANN model which was constructed using the above numerical features only.  Figure 4 shows the proposed architecture for the model. The experiment was then repeated three times and the results were averaged. It can be seen in Table 9 that the proposed model achieved a slightly higher weighted *F*1-score of 0.59 compared to the baseline *F*1-score of 0.58. This difference could be attributed to the impact of the text feature in our proposed model considering that the baseline model did not incorporate text features whereas our proposed model had both text and numerical features. Since the difference between the two models is very small, it implies that the contribution of the students' reviews to grades prediction is minimal. From Table 9, the proposed model achieved a weighted average score of 0.82 in predicting *F*-grades.

## 4. Discussion

The aspect-term extraction component automates the process of mining explicit and implicit aspect terms from students' reviews. Aspect terms, being the specific attributes, that students express their views upon, requires mechanisms for efficiently mining them. After experimenting with LSTM-CRF, GRU-CRF, and Bi-LSTM-CRF models using the BIO tagging labelling scheme, the Bi-LSTM-CRF classifier achieved the highest micro- and macro-*F*1-scores of 0.67 and 0.66, respectively. Thus, the Bi- LSTM-CRF classifier can accurately predict 67% of aspect terms in students' reviews and in comparison, with state-of-the-art models for aspect-term extraction, the model performed competitively well. Results reported in a study by [23] on aspect-term extraction using Bi-LSTM and CRF models achieved an *F*-measure score of 44.49% on a Hindi dataset. Zschornack Rodrigues Saraiva et al., [29] experimented with the task of aspect-term extraction on restaurant and laptop datasets using a deep learning classifier together with POS vectors and word dependencies as features. For the 2016 restaurant dataset, they achieved an *F*1-score of 73.64% and 81.47% for the 2014 laptop dataset. POS and word dependencies features were significant in the increase in the performance.

Once the aspect terms were extracted, the aim was to categorize students' reviews into predefined education domain aspect categories. With the help of education domain experts, twelve aspect categories that were frequent in the dataset were defined. These categories are assessment, course content, course delivery approach, course practical hours, course lecture hours, course tutorial hours, experiential learning, teaching and learning environment, teaching and learning resources, and general. After defining the aspect categories, four variants of RNNs: LSTM, Bi-LSTM, GRU, and Bi-GRU were developed. The different classifiers' performance was within the same range but GRU had a slightly higher performance of 0.78 compared to the rest of the models. In this regard, this study recommends the use of GRUs classifiers for categorizing students' reviews, especially for smaller datasets.

The third part of this study focused on mining sentiment polarity from the students' reviews. The goal was to classify the reviews into either positive or negative sentiment classes. Positive reviews included feedback where students expressed satisfaction with aspects being reviewed, whereas negative feedback consisted of reviews where students were currently not pleased and suggested improvements. Using a manually labelled dataset, experimentation was performed with Bi-GRU and an LSTM baseline model suggested by [19]. The proposed Bi-GRU model achieved an overall weighted *F*1-score of 0.96 compared to the baseline of 0.94. This good performance can be attributed to the bidirectional processing capabilities of Bi-GRUs. With the assumption that the twelve aspect categories were mutually exclusive in that a review could only belong to one aspect category, this implicitly implied that the sentiment polarity score assigned to a review also applies to the aspect category to which that review belongs.

The final component of this study is the grades prediction component. This component combined students' text reviews and numerical features to predict student's grade where the grade is in the set {A, B, C, D, F}. The Bi-LSTM-ANN neural network and evaluated it against an ANN baseline. The Bi-LSTM-ANN neural network was fed with both text reviews and numerical features. The results showed an overall weighted *F*1-score of 0.59 for the Bi-LSTM-ANN and 0.57 for the baseline model. It can be inferred from these results that the text feedback slightly contributes to a student's academic performance. This claim is further supported by the fact that after performing a heatmap variable selection on our entire dataset variables, the sentiment polarity score variable showed a 0.11 correlation score with the target variable and course final grade. Indeed, there exists a relationship between students' module feedback and final grades, but the relationship is weak as revealed from the heatmap. Since the purpose of the grades prediction model is to identify students at risk of failing certain modules, we treated students predicted to obtain “*F*” grades as those requiring interventions to perform better in the final examinations. The proposed model achieved an *F*1-score of 0.82 in predicting students likely to score “*F*” grades. From the confusion matrix metrics, it can be shown that out of 29 true “*F*” grades, the model accurately predicts 20 of them. This shows that the proposed model is quite promising in predicting the students at risk of failing.

### 4.1. Limitations

The limitations for this study was the lack of available open-source students' reviews dataset. In the future, the authors look forward to evaluating the models with different datasets. For the task of aspect-category identification, it was assumed that the aspect categories were mutually exclusive. In future studies, the authors plan to explore possibilities of dealing with reviews belonging to multiple categories considering that in practice some reviews belong to more than one category.

## 5. Conclusion

The aim of this work was to use deep learning models for aspect-based sentiment analysis of students' reviews about the modules they have studied as well as a grade prediction model in order to predict students are at risk of failing. The accuracy of the models has been evaluated and compared with existing works. A list of aspect categories has been based with the help of domain experts. The framework can be easily replicated for any higher education institution having student reviews in English. The deep learning models used performed reasonably well compared to the state of the art. The work proposed in this paper can help to address problems faced by educational institutions in processing reviews obtained from students. In this competitive era, institutions need to stand out by providing the best student experience and quality education. The ability to automatically process student feedback will allow institutions to become more proactive and addressing issues promptly. With regards to grades prediction, it is in the interest of both students and institutions to keep the level of failure to a minimum. Undoubtedly, deep learning technologies can be leveraged to help educational institutions in this challenging task.

## Figures and Tables

**Table 1:** Phrases associated with aspect categories.

Aspect categories	Key phrases	Train	Test
Assessment	Assignments, examinations marking, and questions	54	17
Course^1^ Delivery Approach	Delivery pace, concepts explanations, clear examples, module delivery, presentation, teaching style, students' participation in class, and interactive learning	198	48
Course content	Curriculum update, adding or removing some topics, syllabus, and workload	28	12
Course general	Review about the course in general	93	13
Course tutorials	Tutorial hours, tutors, and time for tutorials	27	29
Course practical hours	Practical, hands-on practice, laboratory sessions, and practice time	65	15
Course lecturer hours	Lecture hours, time, credit hours, extra hours, and teaching hours	73	14
Course management	Lecturer recruitment, lecturer allocation, learning starting time, coordination of lecturers and students, and course delivery setup, e.g., should the module be taught together with students from other programmes taking the same module?	55	10
Experiential learning	Field trips, academic visitations, industrial visitations, guest lecturers, and experts' seminars	68	11
Professional competencies	Lecturer missing classes regularly, commitment, students' intimidations, friendly learning environment, lecturer behavior, attitude, instructor area of specialization, qualification, and passion	37	9
Teaching and learning resources	Laboratory equipment, books availability in library, and computers	89	18
Teaching and learning environment	Classroom conditions	75	10
Total		862	211

^1^ It is to be noted that course and module are same.

**Table 2:** Tagging an example sentence.

Sentence: “the classroom size is too small”	
Word	Tag
the	O
classroom	B
size	I
is	O
too	O
small	O

**Table 3:** Example of B-tagging, I-tagging, and O-tagging.

Tag	Description
B	The first word of an aspect term, found at the beginning
I	Inside of an aspect term, that is, the word is part of an aspect term
O	Outside of aspect term words

**Table 4:** Training and testing dataset for the aspect-term extraction model.

	Train	Test
Number of reviews	903	208
B-tag	847	186
I-tag	593	144
O-tag	4040	1018

**Table 5:** Precision (*P*), recall (*R*), and *F*1-score (*F*1) for aspects-term extraction models.

	LSTM-CRF			GRU-CRF			Bi-LSTM-CRF		
	*P*	*R*	*F*1	*P*	*R*	*F*1	*P*	*R*	*F*1
B-aspect	0.69	0.69	0.69	0.61	0.70	0.65	0.74	0.74	0.74
I-aspect	0.66	0.24	0.35	0.62	0.38	0.47	0.62	0.46	0.53
Micro avg	0.69	0.53	0.60	0.61	0.58	0.60	0.70	0.64	0.67
Macro avg	0.68	0.53	0.57	0.61	0.58	0.59	0.70	0.64	0.66

**Table 6:** Precision (*P*), recall (*R*), and *F*1-score (*F*1) for models developed for the aspect-term identification model.

Aspect categories	LSTM			GRU			Bi-GRU			Bi-LSTM		
	*P*	*R*	*F*1	*P*	*R*	*F*1	*P*	*R*	*F*1	*P*	*R*	*F*1
Assessment	0.93	0.82	0.87	0.93	0.82	0.87	0.88	0.88	0.88	0.94	0.88	0.91
Content	0.67	0.33	0.44	1.00	0.42	0.59	1.00	0.42	0.59	1.00	0.42	0.59
Delivery approach	0.75	0.81	0.78	0.70	0.83	0.76	0.67	0.81	0.74	0.67	0.79	0.72
General	0.73	0.85	0.79	0.79	0.85	0.81	0.58	0.85	0.69	0.73	0.85	0.79
Lecture hours	0.92	0.73	81	0.83	1.00	0.91	1.00	0.73	0.85	0.92	0.80	0.86
Management	0.71	0.50	0.59	0.56	0.50	0.53	0.75	0.60	0.67	0.54	0.70	0.61
Practical hours	0.82	0.95	0.88	0.86	0.95	0.90	0.86	0.95	0.90	0.90	0.95	0.92
Tutorials	1.00	0.83	0.91	1.00	0.76	0.86	1.00	0.83	0.91	1.00	0.69	0.82
Experiential learning	1.00	0.91	0.95	0.92	1.00	0.96	1.00	1.00	1.00	0.92	1.00	0.96
Professional competencies	0.56	0.56	0.56	0.75	0.33	0.46	0.67	0.22	0.33	0.50	0.22	0.31
Teaching and learning environment	0.53	1.00	0.69	0.57	0.80	0.67	0.53	0.90	0.67	0.62	1.00	0.77
Teaching and learning resources	0.70	0.78	0.74	0.70	0.78	0.74	0.82	0.78	0.80	0.70	0.78	0.74
Accuracy			0.78			0.79			0.78			0.77
Macroaverage	0.78	0.76	0.75	0.80	0.75	0.75	0.81	0.75	0.75	0.79	0.76	0.75
Weighted average	0.80	0.78	0.78	0.81	0.79	0.78	0.81	0.78	0.78	0.80	0.77	0.77

**Table 7:** Results for sentiment polarity using Bi-GRU.

	Run 1			Run 2			Run 3			Run 4		
	*P*	*R*	*F*1	*P*	*R*	*F*1	*P*	*R*	*F*1	*P*	*R*	*F*1
Negative	0.96	0.99	0.98	0.96	0.99	0.98	0.97	0.98	0.98	0.96	0.99	0.98
Positive	0.96	0.81	0.88	0.93	0.81	0.86	0.90	0.84	0.87	0.96	0.77	0.86
Accuracy			0.96			0.96			0.96			0.96
Macroaverage			0.93			0.92			0.92			0.92
Weighted average			0.96			0.96			0.96			0.96

**Table 8:** Comparison of proposed approach in this work with state-of-the-art performance for sentiment polarity prediction.

Approaches	Source	No. polarity categories	Precision	Recall	*F*1-score	Accuracy
SVM	[28]	3	0.91	0.88	0.91	0.97
NB	[28]	3	0.70	0.70	0.71	0.89
CNB	[28]	3	0.78	0.87	0.89	0.93
ME	[28]	3	0.92	0.82	0.92	0.87
LSTM + domain embedding	[19]	3	0.88	0.85	0.86	—
WS-LSTM + glove	[21]	3	—	—	0.93	—
WS-CNN + glove	[21]	3	—	—	0.91	—
Baseline model	This study	2	0.94	0.94	0.94	0.94
Proposed model	This study	2	0.96	0.96	0.96	0.96

**Table 9:** Comparison of proposed approach in this work with state-of-the-art performance for sentiment polarity.

	Bi-LSTM-ANN			Baseline		
	*P*	*R*	*F*1	*P*	*R*	*F*1
A	0.96	0.59	0.73	0.78	0.74	0.76
B	0.44	0.82	0.58	0.45	0.54	0.49
C	0.47	0.50	0.48	0.33	0.46	0.38
D	0.38	0.22	0.28	0.50	0.30	0.37
F	1.00	0.69	0.82	1.00	0.76	0.86
Accuracy			0.59			0.57
Macro average	0.65	0.56	0.58	0.61	0.56	0.57
Weighted average	0.66	0.59	0.59	0.61	0.57	0.58

## Data Availability

The data and materials used to support the study are available from the corresponding author upon request.
